# The Csr system regulates genome-wide mRNA stability and transcription and thus gene expression in *Escherichia coli*

**DOI:** 10.1038/srep25057

**Published:** 2016-04-26

**Authors:** Thomas Esquerré, Marie Bouvier, Catherine Turlan, Agamemnon J. Carpousis, Laurence Girbal, Muriel Cocaign-Bousquet

**Affiliations:** 1 Université de Toulouse, INSA, UPS, INP, LISBP, 135, avenue de Rangueil, 31077 Toulouse, France; 2 INRA, UMR792 Ingénierie des systèmes biologiques et des procédés, 31400 Toulouse, France; 3 CNRS, UMR5504, 31400 Toulouse, France; 4 Laboratoire de Microbiologie et Génétique Moléculaires, UMR5100, Centre National de la Recherche Scientifique et Université Paul Sabatier, 118 Route de Narbonne, 31062, Toulouse, France

## Abstract

Bacterial adaptation requires large-scale regulation of gene expression. We have performed a genome-wide analysis of the Csr system, which regulates many important cellular functions. The Csr system is involved in post-transcriptional regulation, but a role in transcriptional regulation has also been suggested. Two proteins, an RNA-binding protein CsrA and an atypical signaling protein CsrD, participate in the Csr system. Genome-wide transcript stabilities and levels were compared in wildtype *E. coli* (MG1655) and isogenic mutant strains deficient in CsrA or CsrD activity demonstrating for the first time that CsrA and CsrD are global negative and positive regulators of transcription, respectively. The role of CsrA in transcription regulation may be indirect due to the 4.6-fold increase in *csrD* mRNA concentration in the CsrA deficient strain. Transcriptional action of CsrA and CsrD on a few genes was validated by transcriptional fusions. In addition to an effect on transcription, CsrA stabilizes thousands of mRNAs. This is the first demonstration that CsrA is a global positive regulator of mRNA stability. For one hundred genes, we predict that direct control of mRNA stability by CsrA might contribute to metabolic adaptation by regulating expression of genes involved in carbon metabolism and transport independently of transcriptional regulation.

Organisms have developed multiple mechanisms of gene expression regulation to adapt their physiology and metabolism to changing environment in order to compete with other species. At any time in the adaptation process, the intracellular level of an mRNA is the direct result of gene expression regulation. For each gene, the balance of two independent cellular processes, synthesis and degradation of mRNA determines mRNA quantity. These two levels were previously demonstrated to contribute to adaptation processes in various organisms^1^,^2^,^3^,^4^.

At the genome-wide scale, global regulators allow coordinated expression control of a large set of genes after sensing environmental changes. Most of the known global regulators in Bacteria are transcriptional regulators (CRP, Fis, etc…) with well described mechanisms of action. They activate or repress initiation of mRNA transcription by the RNA polymerase, and control global metabolism such as carbon uptake and respiration^5^,^6^. Only a few global regulators act at the post-transcriptional level on mRNA stability. One example is the RNA binding protein Hfq, homolog of the Sm and Lsm proteins that form the core of splicing and mRNA degradation complexes in eukaryotes. Hfq facilitates base-pairing interactions of regulatory noncoding small RNAs (sRNAs) on multiple target mRNAs^7^ to destabilize mRNAs and regulate key bacterial metabolic pathways^8^,^9^.

The Csr/Rsm (carbon storage regulator/repressor of secondary metabolites) system is a multi-component global regulatory system, well conserved in Bacteria, that controls gene expression of many important cellular functions. The Csr system represses glycogen metabolism, gluconeogenesis, biofilm formation and quorum sensing while it activates glycolysis, cell motility, virulence and pathogenesis as demonstrated in γ-Proteobacteria such as *Escherichia, Salmonella, Erwinia, Pseudomonas* or *Vibrio*^10^. It is composed of two proteins, CsrA, an RNA binding protein, and CsrD, a putative signaling protein, and two regulatory sRNAs, CsrB and CsrC^11^. The Csr system is mainly known for its post-transcriptional role in mRNA stability. In *E. coli* K-12 strains, mRNA binding of CsrA has been demonstrated to destabilize four transcripts (*glgCAP, pgaABCD, ydeH* and *ycdT*)^12^,^13^,^14^,^15^ and to stabilize one (*flhDC*)^16^. The CsrD protein stimulates the degradation of CsrB and CsrC^17^. An originality of the Csr system is to combine multi-level regulation acting at the post-translational level but also at the transcriptional level. Besides its role in mRNA stability, the Csr system is indeed involved in post-translational regulation through the CsrA-dependent activation of enzyme activity^18^. The CsrA activity is itself regulated via sequestration by the sRNA CsrB, CsrC and McaS^19^,^20^,^21^,^22^. At the transcriptional level, CsrA interacts with the global stringent response^23^ and the BarA/UvrY two-component signal transduction system^24^,^25^, two major actors in regulation of *E. coli* gene expression. The role of CsrD at the transcriptional level is not yet fully understood. Although CsrD contains GGDEF/EAL domains, CsrD is not directly involved in c-di-GMP metabolism^17^,^26^. However, CsrD was demonstrated to regulate expression of a transcriptional regulator CsgD via mechanisms not yet fully elucidated^26^.

How the Csr system combines transcriptional and post-transcriptional regulation at the genome-wide scale to control major physiological functions is not yet determined. There are a limited number of genomic studies of the Csr system, and they all focused on CsrA. Lists of CsrA targeted genes were provided by mRNA-protein interaction analysis^23^, bioinformatics tools^27^,^28^ and with transcriptomic analysis^15^. When changes in mRNA amounts were identified by transcriptomic analysis in response to *csrA* overexpression^15^, measurement of mRNA stability was not simultaneously performed impeding to discriminate between transcriptional and post-transcriptional regulations.

Here we have elucidated the regulation networks of CsrA and CsrD at both the transcriptional and post-transcriptional levels. We determined and compared genome-wide data of mRNA quantity and mRNA stability in a wild type MG1655 strain, in a strain containing a partial deletion of *csrA*, MG1655(*csrA51*), and in a strain deleted of *csrD*, MG1655(Δ*csrD*). Using an integrative approach, we show that in our experimental condition CsrA and CsrD are both involved in massive transcriptional regulation whereas genome-wide regulation of mRNA stability is only dependent on CsrA. We discuss these results in terms of direct and indirect effects, and show how the direct control of mRNA stability by CsrA contributes to metabolic adaptation.

## Results

### Construction and growth of the MG1655(*csrA51*) and MG1655(Δ*csrD*) mutant strains

The *csrA* gene is essential for cell viability in *E. coli* K-12 strains grown on glycolytic sources^29^,^30^. A hypomorphic mutant corresponding to a transposon insertion into the *csrA* gene is viable and has been used in numerous studies of the role of CsrA^31^,^32^,^33^,^34^. CsrA is a dimeric 61 amino acid RNA binding protein^35^. The transposon insertion results in the production of a 50 amino acid protein deleted of the C-terminal dimerization domain^36^. In order to create isogenic strains in the MG1655 background, we engineered the MG1655(*csrA51*) strain by inserting a stop codon at position 51. In this construct, the endogenous transcription termination signal downstream of the stop codon is conserved. As expected^36^, the MG1655(*csrA51*) strain produced much more glycogen than the MG1655 strain and this phenotype is abolished by complementation with an ectopic copy of the wild type *csrA* (Supplementary Fig. S1). The mutant strain also displayed significantly less motility and more biofilm formation (Supplementary Fig. S1) as expected^14^,^16^. Measurement by Northern Blot of CsrB and CsrC levels in the MG1655(*csrA51*) strain showed the absence of expression of the two sRNAs (Supplementary Fig. S1) confirming the role of CsrA in transcriptional activation of CsrB and CsrC^24^. All these results confirmed that the activity of the C-terminal truncated variant of CsrA was drastically diminished in our MG1655(*csrA51*) strain^10^.

**Figure 1:**
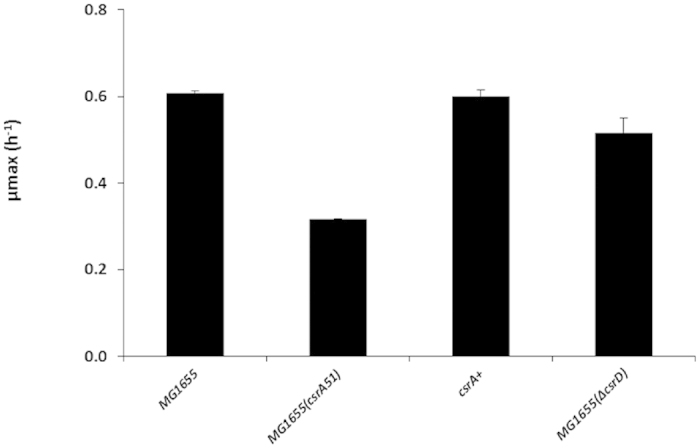
Maximal growth rates (μ_max_) of the MG1655, MG1655(*csrA51*), MG1655(*csrA51*) complemented with a plasmid copy of the wild type *csrA* gene (*csrA*+), and MG1655(Δ*csrD*) strain in M9 minimal medium supplemented with glucose.

The MG1655(Δ*csrD*) strain was constructed by total deletion of the *csrD* gene in *E. coli* MG1655. When cultured in batch in M9 minimal medium supplemented with glucose, MG1655(*csrA51*) cells exhibited a two-fold reduced maximal growth rate (μ_max_ = 0.31 ± 0.01 h^−1^) compared to wild type (μ_max_ = 0.63 ± 0.01 h^−1^) (Fig. 1); normal growth was restored by complementation with an ectopic copy of the wild type *csrA* gene (Fig. 1). In contrast, the maximal growth rate was only slightly affected in MG1655(Δ*csrD*) (0.52 ± 0.08 h^−1^) compared to the MG1655 strain (Fig. 1). To eliminate the growth rate effects on mRNA half-lives and levels^3^ and to permit comparison between strains, the MG1655, MG1655(*csrA51*) and MG1655(Δ*csrD*) strains were cultivated in continuous culture at an equivalent growth rate of 0.10 h^−1^. The *csrA51* or Δ*csrD* mutations did not affect the macro kinetics behavior of the strains (Table 1). Glucose (3 g/l) was totally consumed and no acetate was produced. A slightly higher biomass yield was measured in the MG1655(*csrA51*) strain. The *csrA51* mutation was associated with a 5-fold higher intracellular glycogen content compared to the MG1655 strain (Table 1).

### Effect of the *csrA51* and Δ*csrD* mutations on mRNA stability

**Figure 2:**
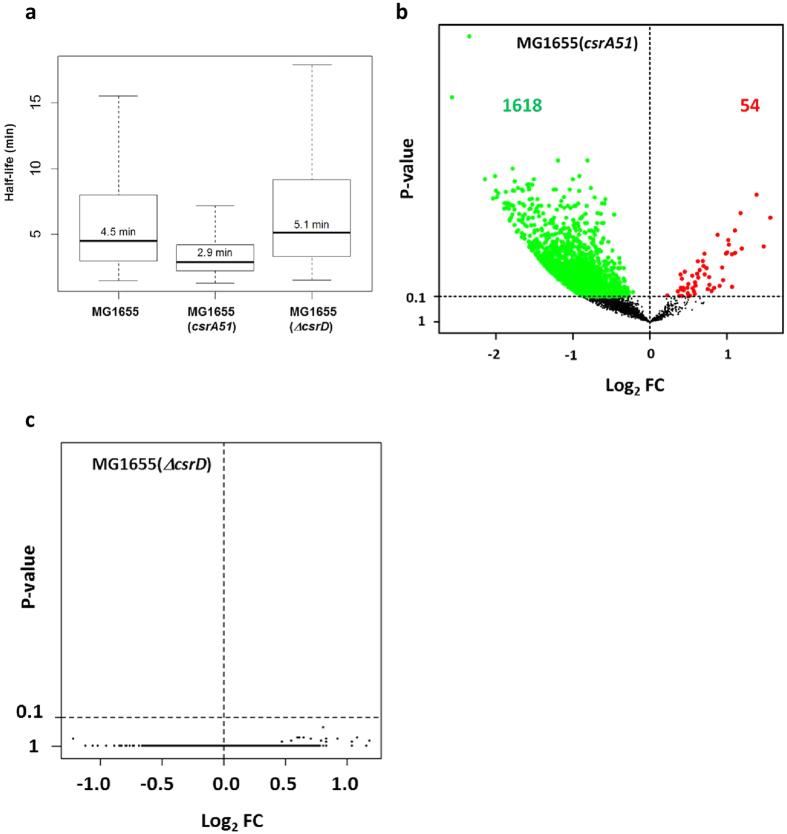
Effect of the *csrA51* and Δ*csrD* mutations on mRNA half-life. (**a**) Box plots of transcript half-life for the MG1655, MG1655(*csrA51*) and MG1655(Δ*csrD*) strains (n = 3351 mRNAs). Values are separated into four quartiles by horizontal bars. The central bar (in the middle of the rectangle) represents the median value, which is given above the bar. VolcanoPlot of the log_2_ fold change (Log_2_ FC) of mRNA half-lives (**b**) between the MG1655(*csrA51*) and MG1655 strains (n = 3028 mRNAs) and (**c**) between the MG1655(Δ*csrD*) and MG1655 strains (n = 3333 mRNAs). A P-value ≤ 0.1 was required for fold change significance (above the horizontal dashed line). The significantly stabilized mRNAs in the mutant strain compared to the MG1655 strain are colored in red whereas the significantly destabilized mRNAs are colored in green.

CsrA has been demonstrated to regulate the stability of a few mRNAs^11^. However, many more putative targets are suggested by interaction study^23^ and bioinformatics analysis^27^,^28^. CsrD is involved in the turn-over of the two sRNA CsrB and CsrC^17^ but its involvement in mRNA stability regulation has not yet been investigated. To explore CsrA and CsrD involvement in mRNA stability regulation, we compared genome-wide measurements of mRNA stability in the MG1655, MG1655(*csrA51*) and MG1655(Δ*csrD*) strains grown in continuous culture at 0.1 h^−1^ (Supplementary Table S1). Transcripts were globally less stable in MG1655(*csrA51*) than in the MG1655 strain, the median half-life decreasing from 4.5 min in the MG1655 to 2.9 min in the mutant strain (Fig. 2a). More precisely, a statistical test (P-value ≤ 0.1) identified 1672 messengers with differential half-life between the MG1655(*csrA51*) and MG1655 strains: 1618 were significantly destabilized in the mutant strain whereas 54 were significantly stabilized (Fig. 2b). Interestingly, these results show a genome-wide involvement of CsrA in mRNA stability regulation and more particularly a massive destabilization of mRNA in the MG1655(*csrA51*) mutant strain.

**Figure 3:**
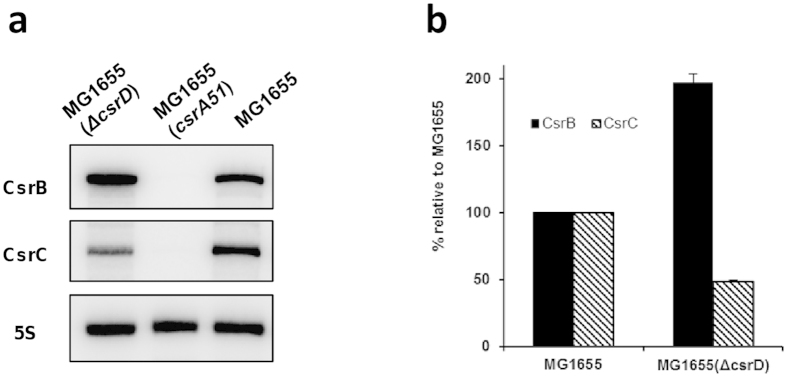
Level of CsrB and CsrC in the MG1655, MG1655(*csrA51*) and MG1655(Δ*csrD*) strains in continuous culture. (**a**) Northern blots of total RNA probed for CsrB and CsrC. (**b**) Hybridization signals quantified on a PhosphorImager. The signals for MG1655 were set to 100%.

The median mRNA half-life in MG1655(Δ*csrD*) was only slightly higher than in the MG1655 strain (Fig. 2a). However, applying the same statistical test used for the MG1655(*csrA51*) strain (P-value ≤ 0.1), no mRNA with a significant variation of stability was found between the MG1655(Δ*csrD*) and MG1655 strains (Fig. 2c) suggesting a small systematic increase in global mRNA stability. This observation is quite surprising since according to the established regulatory network of the Csr system, CsrD is believed to positively regulate CsrA activity via CsrB and CsrC destabilization^17^. Consequently, a destabilization as already observed for MG1655(*csrA51*) strain was expected in the MG1655(Δ*csrD*) strain. In order to understand better this phenotype, we measured by Northern Blot CsrB and CsrC half-lives and levels. As previously shown^17^, CsrB and CsrC sRNAs were strongly stabilized in the MG1655(Δ*csrD*) strain (Supplementary Fig. S2) but their levels were not modified accordingly, suggesting a transcriptional retro-control: indeed the CsrB level was only increased around two fold in our experimental condition whereas the CsrC level was even decreased around two fold (Fig. 3). Since CsrD regulation of CsrB and CsrC turnover depends on the physiological status of the cells^37^, the CsrB and CsrC sRNA stabilization shows that CsrD was active in our growth condition. The opposite regulation of CsrB and CsrC levels is expected to only slightly modify the amount of CsrA sequestrated by the two sRNAs in the MG1655(Δ*csrD*) strain compared to the MG1655 strain. Therefore, CsrA should exhibit a rather similar activity in the two strains. This conclusion was supported at the phenotypic level by the absence of a CsrA-related glycogen response in the MG1655(Δ*csrD*) strain compared to the MG1655 strain. We measured the glycogen content in the MG1655(Δ*csrD*) strain and we did not find an increase of the glycogen content (Table 1). In our growth conditions, the presence/absence of CsrD did not affect mRNA stability neither via CsrD direct targeting nor via CsrA activity.

### Effect of the *csrA51* and Δ*csrD* mutations on mRNA levels

**Figure 4:**
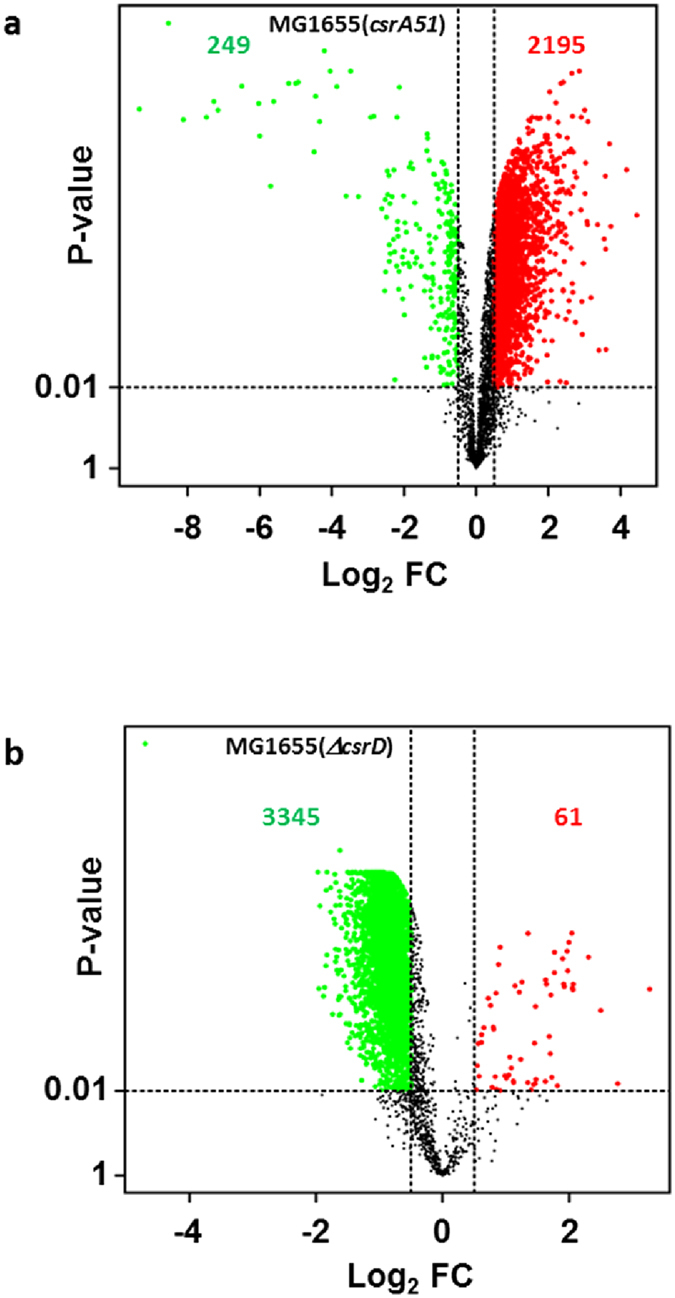
Effect of the *csrA51* and Δ*csrD* mutations on mRNA levels. VolcanoPlot of the log_2_ fold change (Log_2_ FC) of mRNA amounts (n = 4254 mRNAs) (**a**) between the MG1655(*csrA51*) and MG1655 strains and (**b**) between the MG1655(Δ*csrD*) and MG1655 strains. A P-value ≤ 0.01 (above the horizontal dashed line) and a log_2_ FC higher than 0.5 or lower than −0.5 (outside the vertical dashed lines) were required for fold change significance. Between the mutant strain and the MG1655 strain, the significantly up-regulated amounts were colored in red whereas the significantly down-regulated levels were colored in green.

To assess the transcriptional influence of CsrA and CsrD, we first measured the transcriptomic response in MG1655(*csrA51*) and MG1655(Δ*csrD*) mutant strains (Supplementary Table S2). In contrast to the massive destabilization of mRNA in the MG1655(*csrA51*) strain, transcriptomic analyses showed a global up-regulation of mRNA amounts with 2195 mRNAs significantly higher compared to 249 mRNAs significantly lower (Fig. 4a). Up-regulated expressions were mainly related to biogenesis of cell wall and membrane and to signal transduction mechanisms while the down-regulated expressions were related to cell motility and carbohydrate transport and metabolism (Supplementary Table S3). These results are consistent with our phenotypic characterization and the literature (less motility, more biofilm formation and glycogen accumulation). The groups “Flagellum organization” and “Ciliary or flagellar motility” (containing *flhDC* gene, *fli* and *flg* operons) were enriched in the down-regulated mRNA levels correlating with reduced motility^16^,^38^. GO term “Single species biofilm formation” (including *csg* and *ydeH* genes) was enriched in the group of up-regulated mRNA levels but also *yddV, yjjC* and *yliE* mRNAs encoding GGDEF/EAL proteins involved in c-di-GMP metabolism^15^,^33^. All transcripts involved in glycogen synthesis (*glgC, glgB, glgA* and *glgP/Y*) were at least 3-fold up-regulated consistently with the high level of glycogen produced in MG1655(*csrA51*) in our experimental condition (Table 1). Moreover, numerous transcripts whose expressions are known to be controlled by CsrA including *pck, cstA, hfq, relA* and *spoT* have behavior consistent with the literature^23^,^36^,^39^,^40^.

A strong transcriptomic response was observed in the MG1655(Δ*csrD*) strain compared to the MG1655 strain (Fig. 4b). A large majority of the genes were down-regulated (3345/4254) and only 61 were significantly up-regulated. In the MG1655(*csrA51*) strain, a connection between CsrA and CsrD expression is expected due to the negative regulation of *csrD* gene expression by CsrA^15^,^17^. Our results show that *csrD* mRNA quantity was strongly enhanced in the MG1655(*csrA51*) strain (4.6-fold increase). Considering this result, we have compared the transcriptomic responses in the MG1655(*csrA51*) and MG1655(Δ*csrD*) strains. 77% (1878/2444) of differentially expressed mRNAs in MG1655(*csrA51*) were also differentially expressed in Δ*csrD* and for most of them (1653/1878) the regulation was in opposite directions: most of the up-regulated mRNAs in MG1655(*csrA51*) were down-regulated in the MG1655(Δ*csrD*) strain. Altogether these results show that mutations of CsrA and CsrD induce strong but opposite global regulation of mRNA levels and that the transcriptomic response observed in the MG1655(*csrA51*) strain could be at least partially mediated by CsrD.

### CsrA and CsrD are involved in massive transcriptional regulation

To decipher the global roles of CsrA and CsrD at the transcriptional level, we integrated datasets of mRNA stability and mRNA quantity measured in the MG1655, MG1655(*csrA51*) and MG1655(Δ*csrD*) strains. A difference in mRNA concentration between each mutant and the wild type strain can be the consequence of change in mRNA synthesis (transcription), degradation and/or mRNA dilution as cells divide not necessarily at the same rate. In our experiments, all the strains were cultivated at the same growth rate, so the effect of mRNA dilution on gene expression can be eliminated. Therefore, all changes in mRNA level in our study can only be explained by transcriptional and/or mRNA stability regulation.

In the MG1655(Δ*csrD*) strain, no individual transcript stability was significantly modified whereas a negative transcriptomic response was observed for 3345 genes. Therefore, these changes in mRNA levels result mainly from down regulation of transcription. Similarly, in MG1655(*csrA51*), 542 mRNAs were regulated at the mRNA level (positively for 82%), but not in stability. This demonstrates that mutating *csrA* provokes a transcriptional response, which is generally positive. These results show that CsrD and CsrA regulate a large number of genes by acting only at the transcriptional level. Molecular validation of widespread transcriptional action of CsrA and CsrD was provided by transcriptional fusions using a *lacZ* reporter gene. We confirmed higher *lacZ* expression under the control of the *glgB* and *ydeH* promotors in the MG1655(*csrA51*) strain compared to the MG1655 strain, and the lower *lacZ* expression under the control of the *frdA* and *csrC* promotors in the MG1655(Δ*csrD*) compared to the MG1655 strain (Supplementary Table S4).

To go deeper in the transcriptional regulation, we searched for changes in mRNA levels of genes related to the functional category “Transcription, DNA-dependent” in both MG1655(Δ*csrD*) and MG1655(*csrA51*), compared to the MG1655 strain. Interestingly opposite regulation was observed in the mutant strains, as genes encoding protein involved in transcription were down-regulated in the MG1655(Δ*csrD*) strain but up-regulated in MG1655(*csrA51*) (Table 2). These genes correspond to RNA polymerase (β and β’ subunits, *rpoB* and *rpoC*), sigma factors (*rpoD, rpoE, rpoH, rpoN, rpoS* and *fecI*), factors involved in the stringent response (*relA, spoT* and *dksA*) and global transcriptional regulators. More precisely, the transcriptional regulators were *crp, cyaA* and *fruR*/*cra* for regulating the carbon flow, *fnr* and *arcA* involved in regulation of the aerobic/anaerobic metabolisms and *ihfA* and *ihfB* encoding the IHF factor involved in chromosomal structure. Taken together with the up-regulated expression of *csrD* mRNA in the MG1655(*csrA51*) strain, these results support the conclusion that CsrD-mediated transcriptional regulation contributes to the transcriptional response observed in MG1655(*csrA51*).

### CsrA is a global positive regulator of mRNA stability

**Figure 5:**
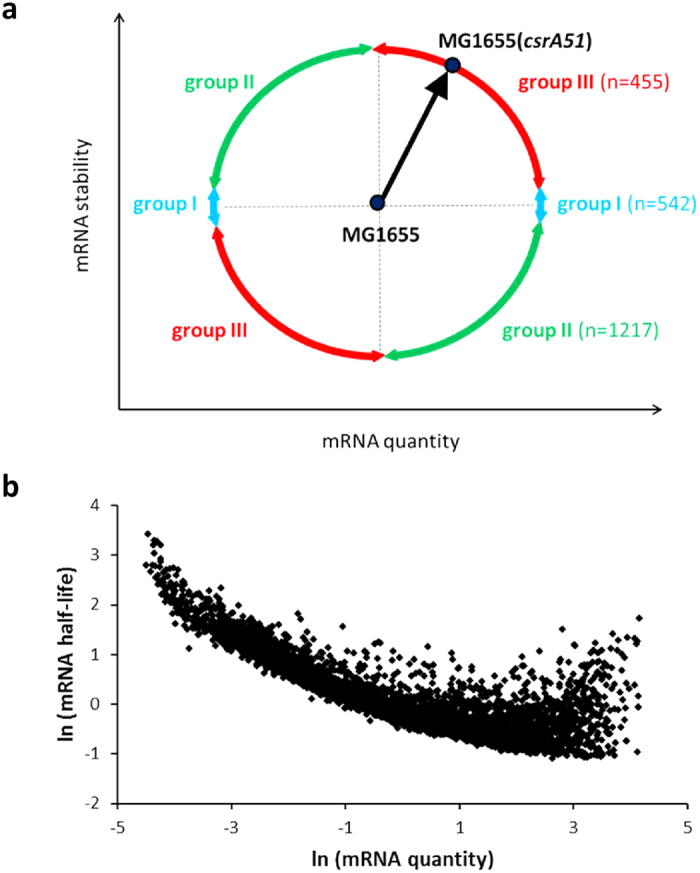
(**a**) Variations in mRNA stability and quantity between the strains MG1655(*csrA51*) and MG1655. mRNAs with variation in level but not in stability in MG1655(*csrA51*) compared to MG1655 are in group I, those with variations in stability and quantity in opposite directions are in group II, and those with variations in stability and quantity not in opposite directions are in group III. (**b**) Plots of transcript half-life (in min) as a function of transcript quantity (in arbitrary units per mg of dry cell weight) in the MG1655 strain (n = 3074 mRNAs) and MG1655(*csrA51*) (n = 4098 mRNAs). All the values were log-transformed and centered. The Pearson correlation coefficient is −0.81 with a P-value < 2.2E^−16^.

To decipher the precise role of CsrA in large scale mRNA stability regulation, we considered variations in both mRNA stability and concentration between MG1655(*csrA51*) and MG1655. Stability and level variations in MG1655(*csrA51*) compared to MG1655 were schematically classified in three groups (Fig. 5a and Supplementary Table S5). Group I corresponded to a variation in mRNA amount not associated with a variation in stability. This group contained the above mentioned 542 mRNAs whose levels were under transcriptional control. In the two other groups, both the stability and quantity differed in MG1655(*csrA51*) compared to MG1655. In group II (n = 1217), stability and quantity varied in opposite direction (lower stability associated with a higher amount in MG1655(*csrA51*) compared to MG1655 or vice versa) whereas group III (n = 455) included mRNAs exhibiting stabilization with similar or a higher level and destabilization with a similar or lower level.

In a previous study, we have demonstrated in *E. coli* at the genome-wide scale that the quantity of an mRNA is the major determinant of its stability^41^. Plotting mRNA half-life as a function of its amount in MG1655(*csrA51*) and MG1655 showed a strong negative correlation (Fig. 5b). This indicates that for a given mRNA, a change in stability between MG1655(*csrA51*) and MG1655 can potentially be linked to a change in concentration. In other words, mRNA stabilization in MG1655(*csrA51*) may result from a lower mRNA amount whereas a destabilization may be due to a higher amount. This relationship has been validated at the molecular level (Nouaille *et al*., manuscript in preparation). Therefore, for the 1217 transcripts of group II with antagonistic regulation of stability and quantity in MG1655(*csrA51*), the regulation of mRNA stability could correspond to a direct effect of CsrA on mRNA stability or an indirect effect due to change in mRNA level depending on transcription regulation. The regulation of stability of these mRNAs by CsrA is thus complex due to overlapping direct and indirect effects. Most of the mRNAs (1215/1217) were destabilized with up-regulated level and functional classes such as “DNA replication” and “signal transduction” were enriched in this group (Supplementary Table S5). The presence of a consensus CsrA binding site RUACARGGAUGU^27^ was investigated in the −100/+100 nt region (+1 corresponding to the start codon) of the 1217 transcripts. 635 displayed at least one putative CsrA binding site.

For the 455 mRNAs of group III with stability and quantity varying in the same directions, variation in half-life cannot be explained by variation in amount according to the correlation shown in Fig. 5b. Consequently, it is likely that their stability is directly controlled by CsrA; these mRNAs were therefore predicted as putative direct post-transcriptional targets of CsrA. Many were novel CsrA targets, since cross-comparisons of our list with previous studies showed that only 10 and 93 were already *in silico* predicted^28^ or shown to co-purify with CsrA^23^, respectively (Supplementary Table S6). As previously, the presence of a consensus CsrA binding site was investigated in the 455 transcripts. We found that 180 mRNAs displayed unique putative CsrA binding site while 78 had at least 2 sites with hot spot localization in the 5′UTR region (Supplementary Table S6). Taken together, more than 65% (297/455) were predicted to experimentally bind CsrA^23^ and/or to have a putative CsrA binding site. These results suggest that most of the identified putative CsrA direct post-transcriptional targets are likely to interact physically with the CsrA protein although false positive experimental binding^23^ and degenerated CsrA binding motif cannot be totally excluded. We found that more than 88% (403/455) of the CsrA direct post-transcriptional targets were destabilized in the MG1655(*csrA51*) strain (Supplementary Table S6). Together with the results shown in Fig. 2b, our work suggests that CsrA has an important role in the stabilization of a large number of messengers.

### Metabolic impact of the direct regulation of mRNA stability by CsrA

The predicted direct regulation by CsrA of the stability of 455 mRNAs is not expected to contribute in the same way to the control of metabolic adaptation. For 356 mRNAs, a change in stability between the two strains was not associated with a significant change in mRNA level, which is generally required to provoke protein concentration variations. For these mRNAs, stability regulation was counteracted by transcription regulation. These mRNAs were mainly related to translation and carbohydrate transport and metabolism (Supplementary Table S5). On the contrary, for 99 transcripts (Supplementary Table S7), changes in stability were associated with changes in mRNA amounts and might therefore contribute to metabolic differences between the two strains.

More precisely, 64 mRNAs were destabilized with down-regulated level whereas 35 were stabilized with up-regulated level between the MG1655(*csrA51*) and MG1655 strains (Supplementary Table S7). Among the destabilized and down-regulated mRNAs in the MG1655(*csrA51*) strain, we found *tpiA* in agreement with a previous result showing that the triose-phosphate isomerase activity was positively regulated by CsrA activity^42^. We also identified the *zwf* transcript, previously reported to co-purify with CsrA^23^, but its stability and amount have never previously been shown to be regulated by the Csr system. Our results clearly showed down-regulated mRNA stability and quantity of genes encoding carbohydrate transport (*araF, malE, alsB, idnT, melB, glpF, srlAEB* and *agaVW*) and certain metabolic pathways, e.g. idonate/gluconate metabolic process (*idnDOT*) and galacturonate catabolic process (*uxaCA)* in MG1655(*csrA51*) (Supplementary Table S5). mRNA stability and level of a large number of metabolic enzymes (*aldA, ldhA, udp, yfaU, hdhA, glpK, dmlA, gloB, aes, fdhF, speB, tpx, thiH, ydiB, ilvC, pptA, ebgC* and *frlB*) were found to be also negatively regulated in MG1655(*csrA51*).

In the stabilized and up-regulated mRNAs in the MG1655(*csrA51*) strain, several genes belonged to the glycogenesis and gluconeogenesis pathways: *maeA, maeB, pck, gapA, pgm* and *glgB* (Supplementary Table S5). The expression and/or activity of some of these genes/proteins were known to be negatively controlled by CsrA^36^,^42^. Our results provide a better understanding of the CsrA effect on these targets, the regulator acting at the level of both mRNA stability and quantity. Expression (stability and level) of several genes of the Krebs cycle and the amino acid metabolism were found to be upregulated in the MG1655(*csrA51*) strain: *acnB, fumA, sdhC, leuB, leuC, glnA* and *asd*. We observed that the messengers of the mannose PTS permease (*manXYZ*), of the pyridine nucleotide transhydrogenase (*pntAB*) and of the fatty acid synthesis (*fabD* and *fabF*) were stabilized and exhibited higher level in the MG1655(*csrA51*) strain. The *csrA51* mutation also stabilizes and increases the amount of mRNAs related to transcription (*rpoD* encoding the vegetative sigma factor σ^70^ of RNA polymerase) and to translation (ribosomal *rplP, rplN* and *rpsS* transcripts). This and the accumulation of glycogen (Table 1) could partially explain the slightly higher biomass yield in the MG1655(*csrA51*) strain compared to the MG1655 (Table 1).

For the 99 mRNAs whose level and stability were regulated in the MG1655(*csrA51*) strain, we examined the contribution of stability regulation to the control of the mRNA amount. Regulatory coefficients were calculated between MG1655(*csrA51*) and the MG1655 strain. This quantifies the respective effects of transcription regulation and stability regulation on mRNA amount^3^. For a large majority of these mRNAs (85/99), the regulation of stability predominantly controlled mRNA level. This indicates that it was a change in stability that provoked differential mRNA amount in the MG1655(*csrA51*) strain and this is true even if transcription regulation is present; we called this type of regulation degradational control of the mRNA quantity (Supplementary Table S7). Altogether these results provide the first demonstration of how direct regulation (positive/negative) of mRNA stability by CsrA regulates a hundred mRNA amounts independently of transcription regulation and therefore contributes to control the global metabolism.

## Discussion

**Figure 6:**
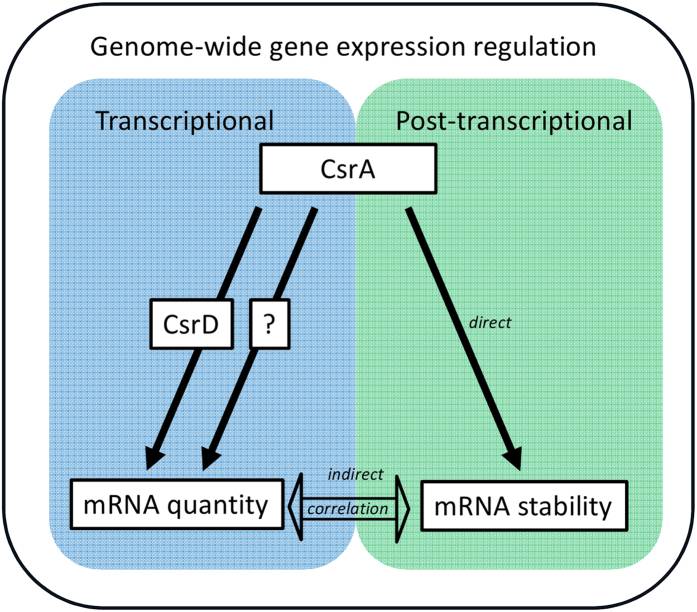
Scheme of the connections described in this study between the transcriptional and post-transcriptional regulatory networks, CsrA and CsrD, which are involved in the genome-wide regulation of gene expression. Filled arrows represent a connection type “act on” while the empty arrow represents a correlation.

In this study, we applied for the first time an integrative biology approach to decipher the transcriptional and post-transcriptional roles of CsrA and CsrD. Although the Csr system is generally believed to be involved in a post-transcriptional response, the protein CsrD was demonstrated here to be involved in a massive positive transcriptional regulation. In contrast, mutating the *csrA* gene induces both transcriptional and post-transcriptional responses. Transcription of more than 500 genes and stability of more than 1600 mRNAs differ between the MG1655(*csrA51*) and MG1655 strains. Opposite transcriptional responses were obtained in the MG1655(*csrA51*) and MG1655(Δ*csrD*) strains. Since we confirmed the strong up-regulation of *csrD* expression in MG1655(*csrA51*), it is likely that the transcriptional response observed in MG1655(*csrA51*) was at least partially mediated by CsrD. The importance of the negative feedback created by CsrA regulation of CsrD expression was previously highlighted in the dynamic response of the Csr system^43^. The time of turning on and off expression of CsrA target proteins was dependent on the CsrD expression level. Our study shows that the regulatory control of the Csr system involves genome-wide transcriptional and post-transcriptional regulation of gene expression connected by CsrA and CsrD (Fig. 6).

Several mechanisms of CsrD action at the transcriptional level can be proposed. This may occur indirectly via transcriptional factors whose expressions are CsrD-dependent. Many transcriptional regulators are down-regulated in the absence of CsrD. The actions of CsrD on transcriptional regulators could involve regulation of enzymes of the c-di-GMP signaling pathway, as shown for CsgD^26^. Another possibility is that CsrD regulates the turnover of sRNAs other than CsrB and CsrC^17^, which in turn control transcriptional regulator expression at the post-transcriptional level. Finally, we cannot exclude a direct transcriptional effect of CsrD on targeted genes through a still unknown mechanism.

A strong negative correlation between mRNA half-life and its amount has been observed here for the whole genome, indicating that as a general trend mRNA stabilization can be due to a decrease in mRNA amount and vice versa. The regulation of mRNA stability by CsrA was complex for the 1217 transcripts with opposite variations of stability and quantity in MG1655(*csrA51*). For these genes, direct effect of CsrA on stability and the potential indirect effect of CsrA on stability due to change in mRNA level cannot not be discriminated (Fig. 6). Nevertheless for more than 400 mRNAs with stability varying in the same direction as mRNA quantity or not associated with mRNA quantity variation, we predicted that CsrA regulates directly their stability. For the majority of these mRNAs, control may be attributed to a physical interaction between CsrA and the mRNA molecule as indicated by the prediction of a putative binding site and/or high throughput binding analysis. For one third of these mRNAs, there was no experimental proof of CsrA binding^23^ and we did not identify any consensus CsrA binding site. Either the sequences of the CsrA binding site were highly degenerated compared to the consensus in these mRNAs or CsrA may act on their stability through other post-transcriptional regulators (such as the RNA-binding protein Hfq) whose expression is CsrA-dependent^39^. About 88% of the half-lives directly regulated by CsrA were shorter in the MG1655(*csrA51*) strain than in the MG1655 strain. This result is surprising because, to date, most of the known targets of CsrA are repressed and/or destabilized, and at the molecular level, examples of repression are more numerous than positive regulation^11^,^13^,^44^,^45^,^46^. Thus, contrary to earlier studies on selected targets, our genome-wide analysis shows that CsrA has a significant role as a positive regulator of mRNA stability. The protective effect of CsrA binding on mRNA by sequestering RNase E cleavage sites, only described until now in the case of *flhDC*^45^, might be common.

In terms of gene expression regulation, for one hundred mRNAs, the direct regulation of mRNA stability by CsrA led to a significant variation in mRNA concentration. We confirmed previously identified metabolic targets of CsrA such as *glgB, pgm, tpiA* and *pck* mRNAs^36^,^42^. In addition, many new direct mRNA targets of CsrA were discovered with a potential metabolic impact via mRNA level changes. Functional analysis of these transcripts underlined that the Csr system is strongly involved in the control of *E. coli* metabolism by regulating expression of enzymes involved in the central carbon metabolism and carbohydrate transport but also in other specific metabolic pathways (syntheses of amino acids and fatty acids). In the MG1655(*csrA51*) strain, mRNA stability regulation favors gluconeogenesis especially anaplerotic reactions (e.g. malic enzyme, PEP carboxykinase), whereas the transport of glycolytic substrates (such as sorbitol, melibiose, gluconate, allose, maltose and arabinose) is down-regulated. Therefore, compared to MG1655, the MG1655(*csrA51*) strain is more likely to efficiently metabolize gluconeogenic substrates. Consequently, our results provide the first evidence at the genome-wide scale of the role of CsrA-dependent regulation of transcript stability in metabolic adaptation.

Our study provides new insights into the Csr regulatory network. We determined the extent, the nature and the metabolic effect of the CsrD and CsrA regulations. Our results highlight the superimposition at the genome-wide scale of the transcriptional and post-transcriptional controls that likely contribute to the robustness and dynamics of gene expression reprogramming during bacterial adaptation.

## Methods

### Bacterial strains and plasmids

*E. coli* K-12 MG1655 strain (λ^−^ F^−^ *rph*^−1^) was the genetic background for mutation of *csrA* and *csrD* genes. Mutagenesis was carried out using λ red recombination^47^ and primer pair OKT-24/OKT-25. As the *csrA* gene is essential^29^,^30^, the ORF was mutated using the *csrA* mutant obtained by Romeo and coworkers as a model^36^. The MG1655(*csrA51*) strain expresses a truncated variant of CsrA in which 11 amino acids at the C-terminal end of the protein have been deleted. The MG1655(Δ*csrD*) strain has a total deletion of the *csrD* gene, which was obtained from the KEIO collection^29^. The mutations were transferred into *E. coli* K-12 MG1655 wild type background by P1 phage-mediated transduction before removing the antibiotic-resistance cassette by FLP recombination. MG1655(*csrA51*) strain was complemented by a low copy number plasmid containing the wild type *csrA* gene. Briefly, *csrA* was amplified using primer pair OCT-29/OCT-30 and cloned in pSAB11^48^ at *Eco*RI site. To perform gene expression assays, *csrC*-*lacZ, glgB*-*lacZ, frdA*-*lacZ* and *ydeH*-*lacZ* transcriptional fusions were constructed. The transcriptional fusions were cloned in pJYB79 (CmR), a plasmid that was constructed by inserting the omegon kan from pHP45-kan into pACYC184 using the restriction site *Bam*HI and subsequently by cloning at *Pst*I site a PCR amplified fragment containing plac-*lacZ* from *E. coli*. First, a 5′UTR sequence containing a ribosome binding site and a start codon (GAATTCCCGGGGATCCTAAGTAAGTAAGGAGAAAAAAATGGCTGATCCC) was fused to *lacZ* 10^th^ codon. Then, the vector was amplified by PCR with primers MBO-200/-199 and assembled with the promoter regions using In-Fusion® HD cloning kit (Clontech). The promoters regions upstream of the transcription starts of *csrC* (249 nt), *glgB* (200 nt), *frdA* (205 nt) and *ydeH* (421 nt) were amplified on MG1655 genomic DNA with oligonucleotide pairs MBO-137/-138, MBO-203/-204, MBO-207/-208 and MBO-197/-198, respectively. For *glgB, frdA* and *ydeH*, the promoter regions that were chosen using information available on the EcoCyc *E. coli* Database listing previously characterized transcriptional regulators. For *csrC*, the promoter region is as used in Suzuki *et al*.^17^. The strains and plasmids were validated by sequencing. The oligonucleotides are listed in Table S8.

### Culture conditions

For RNA extraction and microarray analysis, cells were grown in continuous culture at 37 °C in M9 minimal medium supplemented with glucose as described^3^. The MG1655, MG1655(*csrA51*) and MG1655(Δ*csrD*) strains were cultured at the same growth rate, μ = 0.10 h^−1^, which corresponds to doubling time of 6.9 h. Each culture was repeated three times to provide independent biological replicates. Biomass was estimated from absorbance at 600 nm (Libra S4, Biochrom): 1 unit of absorbance corresponding to 0.42 g of dry cell weight l^−1^ for the MG1655 strain, 0.27 g of dry cell weight l^−1^ for the MG1655(*csrA51*) strain and 0.44 g of dry cell weight l^−1^ for the MG1655(Δ*csrD*) strain. To determine the maximal growth rate of each strain, the strains were grown in batch culture (identical medium, oxygen concentration, pH and temperature) and the rates were determined in exponential growth phase.

### Sampling and RNA extraction

Sampling and RNA extraction were conducted as described^3^ with additional cell samplings after rifampicin addition at 9, 15, 20 and 30 min.

### Northern Blot

For each sample, 10 μg of total RNA was denatured for 5 min at 95 °C in RNA loading buffer (95% [v/v] formamide, 0.1% [w/v] xylene cyanole, 0.1% [w/v] bromphenol blue, 10 mM EDTA), separated on a 7 M urea/6% polyacrylamide gel at 250 V and transferred to Hybond-XL membranes (GE Healthcare) by electroblotting (1 h, 50V, 4 °C) in 1X TBE. After UV crosslinking, the membranes were hybridized overnight in Roti®-Hybri-Quick (Roth) at 65 °C with [^32^P] body-labeled riboprobes specific of CsrB (primers OCT-50/OCT-51) or CsrC (primers OCT-52/OCT-53) or at 42 °C with a 5S specific [^32^P] end-labeled oligonucleotide (MBO-59). Hybridization with CsrB or CsrC riboprobe is followed by 15 min washes in 2X, 1X and 0.1X SSC/0.1% SDS solutions at 65 °C. Hybridization with 5S oligonucleotide is followed by 15 min washes in 5X, 1X and 0.1X/0.1% SDS solutions at 42 °C. Hybridization signals were quantified on a PhosphorImager (Typhoon Trio – GE Healthcare) with MultiGauge software (Fujifilm).

### Microarray procedures

A double-stranded cDNA synthesis kit (InvitroGen) was used to produce cDNA from 2 μg of total RNA. cDNA (1 μg) was labeled using the one color DNA labeling kit (Nimblegen – Roche) and labeled cDNA (2 μg) was hybridized onto *E. coli* K-12 gene expression arrays (Nimblegen – Roche) for 17 h at 42 °C according to the manufacturers’ instructions. Arrays were washed and then scanned with a MS200 Microarray Scanner (Nimblegen – Roche). The images were analyzed with DEVA 1.2.1 software. Only raw data were used for further analyses. All array procedures were performed by the GeT-Biopuces platform (http://get.genotoul.fr).

### mRNA level and half-life determination

mRNA quantity determination by transcriptomic analysis was conducted as described^3^. Intensity values were multiplied by the total RNA extraction yield (in μg total RNA per mg of dry cell weight) to provide mRNA amount value in arbitrary units per mg of dry cell weight. RNA extraction yields were 13.1 ± 2.2, 8.7 ± 0.9 and 8.9 ± 1.5 μg RNA per mg of dry cell weight for the MG1655, MG1655(*csrA51*) and MG1655(Δ*csrD*) strains, respectively. Differences in mRNA level were evaluated as described in^3^. The P-values were adjusted for multiple testing by the ‘Benjamini and Hochberg’ (BH) false discovery rate method^49^. Differences in mRNA quantity were considered as significant for adjusted P-values lower than 1% and log_2_ fold change (Log_2_ FC) higher than 0.5 or lower than −0.5. mRNA half-life determination was performed as previously described^3^. The statistical significance of differences in half-life was evaluated using the probability value of interaction between time and the type of strain (MG1655, MG1655(*csrA51*) or MG1655(Δ*csrD*)) in a global model of linear regression. A statistical threshold of 10% was used for adjusted P-values by the “BH” false discovery rate method^49^.

### Determination of mRNA level regulatory coefficients

For the selected mRNAs, the coefficient ρ_D_, corresponding to the relative contribution of the mRNA stability regulation in the control of mRNA level was calculated as previously described^3^ using data of mRNA stability and quantity obtained in the MG1655(*csrA51*) and MG1655 strains. Regulatory classes were defined according to the ρ_D_ value: 0 < ρ_D_ < 0.6, mRNA quantity mainly controlled by transcription or controlled by both transcription and mRNA stability; 0.6 < ρ_D_, mRNA quantity mainly controlled by mRNA stability.

### Enrichment methods

R free statistical software (www.r-project.org) was used for enrichment methods. Functional categories enriched in transcript subgroups were determined by a hypergeometric test on data using the Biological Process of Gene Ontology annotation database (GO project; http://www.geneontology.org/). Only GO terms with associated P-value ≤ 0.05 were considered as significant.

### *In silico* research of CsrA binding sites

To determine if an mRNA contained a potential CsrA binding site, the software PatScan was used^50^. The SELEX-derived consensus RUACARGGAUGU was searched in a −100/+100 nt window (+1 corresponding to the start codon) allowing a maximum of 5 mismatches. Subsequently, only sequences that included a conserved GGA motif were considered as a potential CsrA binding site.

### Analytical methods

Glucose and acetate concentrations were measured by HPLC coupled to a refractometer and with UV detection. The device was equipped with a Bio-Rad HPX87H column maintained at a temperature of 48 °C and 5 mM H_2_SO_4_ was used as the eluent, at a flow rate of 0.5 ml min^−1^. Glycogen staining by iodine vapor, biofilm formation by cristal violet staining and motility assays on 0.3% agar plate were carried out as previously described^51^. The intracellular glycogen quantification was done as previously described^52^. Briefly, the cells were lysed to extract the glycogen which was then hydrolyzed by amyloglucosidase into glucose subunits. The glucose subunits were then quantified using glucose oxidase coupled to the colorimetric reagent o-dianisidine dihydrochloride. Three independent experiments were performed for each assay.

The *csrC*-*lacZ, glgB*-*lacZ, frdA*-*lacZ* and *ydeH*-*lacZ* gene expression assays were performed using cells grown in batch culture at 37 °C in M9 minimal medium supplemented with fructose (2.7 g.L^−1^), casamino acids (0.2%) and chloramphenicol (25 μg.ml^−1^). Under these conditions, the MG1655, MG1655(*csrA51*) and MG1655(Δ*csrD*) strains have similar growth rates (0.39 h^−1^, 0.39 h^−1^ and 0.31 h^−1^ respectively). These conditions aimed at mimicking growth in continuous cultures. In exponential growth, the cells were centrifuged, suspended in Tris (6 mM)/Tricarballylate (1.8 mM) buffer pH 7.2 and lysed mechanically with glass beads. β-galactosidase activity was assayed using 2.5 mM orthonitrophenyl-β-galactoside^53^. Total protein was measured by the Bradford method with bovine serum albumin as the protein standard^54^. Three independent experiments were performed for each assay.

## Additional Information

**How to cite this article**: Esquerré, T. *et al*. The Csr system regulates genome-wide mRNA stability and transcription and thus gene expression in *Escherichia coli. Sci. Rep.* **6**, 25057; doi: 10.1038/srep25057 (2016).

## Supplementary Material

Supplementary Information

Supplementary Table S1

Supplementary Table S2

Supplementary Table S3

Supplementary Table S4

Supplementary Table S5

## Figures and Tables

**Table 1:** Macro kinetics behavior and intracellular glycogen content of the strains MG1655, MG1655(*csrA51*) and MG1655(Δ*csrD*) in continuous cultures at 0.1 h^−1^ in M9 minimal medium supplemented with 3 g/l glucose at 37 °C.

Strain	Residual glucose (mM)	Produced acetate (mM)	*q*_*S*_ (g glucose/g of dry cell weight h^−1^)	Y_x/s_ (g of dry cell weight/g glucose)	Intracellular glycogen content (g glucose/g of dry cell weight)
MG1655	ND	ND	0.29 ± 0.03	0.36 ± 0.01	0.034 ± 0.006
MG1655(*csrA51*)	ND	ND	0.26 ± 0.01	0.41 ± 0.02	0.194 ± 0.006
MG1655(Δ*csrD*)	ND	ND	0.29 ± 0.01	0.35 ± 0.01	0.040 ± 0.007

ND: not detected.

**Table 2:** Effect of the Δ*csrD* and *csrA51* mutations on mRNA level of genes involved in the biological process “Transcription, DNA-dependent”.

GeneId	GeneSym	MG1655(Δ*csrD*)		MG1655(*csrA51*)	
		FC	P-value	FC	P-value
b0080	*fruR/cra*	0.50	2.4E^−07^	1.40	1.6E^−05^
b0145	*dksA*	0.53	7.4E^−06^	1.67	3.3E^−05^
b0912	*ihfB*	0.60	8.2E^−06^	1.43	9.9E^−05^
b1334	*fnr*	0.55	3.0E^−05^	1.31	4.1E^−03^
b1712	*ihfA*	0.58	3.0E^−05^	1.30	2.8E^−03^
b2573	*rpoE*	0.51	3.7E^−05^	1.36	4.3E^−03^
b2741	*rpoS*	0.49	4.0E^−05^	1.28	1.9E^−02^
b2784	*relA*	0.53	1.6E^−05^	2.05	1.0E^−05^
b3067	*rpoD*	0.55	1.8E^−07^	1.26	3.5E^−05^
b3202	*rpoN*	0.54	2.5E^−07^	2.06	1.3E^−07^
b3357	*crp*	0.48	4.0E^−07^	1.39	4.1E^−05^
b3461	*rpoH*	0.51	3.0E^−05^	2.28	1.2E^−05^
b3650	*spoT*	0.58	1.2E^−04^	2.31	1.2E^−05^
b3806	*cyaA*	0.53	2.2E^−07^	1.73	4.9E^−07^
b3987	*rpoB*	0.69	5.8E^−07^	1.13	5.5E^−04^
b3988	*rpoC*	0.53	3.7E^−06^	1.14	2.9E^−02^
b4293	*fecI*	0.54	6.6E^−05^	2.13	2.3E^−05^
b4401	*arcA*	0.50	3.3E^−06^	1.16	2.7E^−02^

GeneId corresponds to the gene Blattner number^55^, GeneSym to the gene name, FC to the fold change of mRNA amounts between the mutant strain and the MG1655 strain and P-value is the associated adjusted P-value.
