# Supplementary material for: The Csr system regulates genome-wide mRNA stability and transcription and thus gene expression in Escherichia coli
Source: Sci Rep. 2016 Apr 26;6:25057. doi: 10.1038/srep25057 (PMC4844966; doi:10.1038/srep25057)
Supplement: Supplementary Information [file srep25057-s1.pdf]

Supplementary Information

The Csr system regulates genome-wide mRNA stability and transcription and thus gene expression in *Escherichia coli*

Thomas Esquerré[1,2,3,4], Marie Bouvier[4], Catherine Turlan[4], Agamemnon J. Carpousis[4], Laurence Girbal[1,2,3,*,+], and Muriel Cocaign-Bousquet[1,2,3,+]

[1] Université de Toulouse; INSA, UPS, INP; LISBP, 135, avenue de Rangueil, 31077 Toulouse, France

[2] INRA, UMR792 Ingénierie des systèmes biologiques et des procédés, 31400 Toulouse, France

[3] CNRS, UMR5504, 31400 Toulouse, France

[4] Laboratoire de Microbiologie et Génétique Moléculaires, UMR5100, Centre National de la Recherche Scientifique et Université Paul Sabatier, 118 Route de Narbonne, 31062, Toulouse, France.

* corresponding author: girbal@insa-toulouse.fr

[+] These authors contributed equally to this work

Figure S1. Phenotypic characterization of the MG1655(*csrA51*) mutant strain. (A) Glycogen staining assay in the MG1655, MG1655(*csrA51*) and *csrA*+ strains. (B) Motility and biofilm formation assays in the MG1655 and MG1655(*csrA51*) strains. (C) Level of CsrB and CsrC in the MG1655 and MG1655(*csrA51*) strains in Northern blots of total RNA probed for CsrB and CsrC.

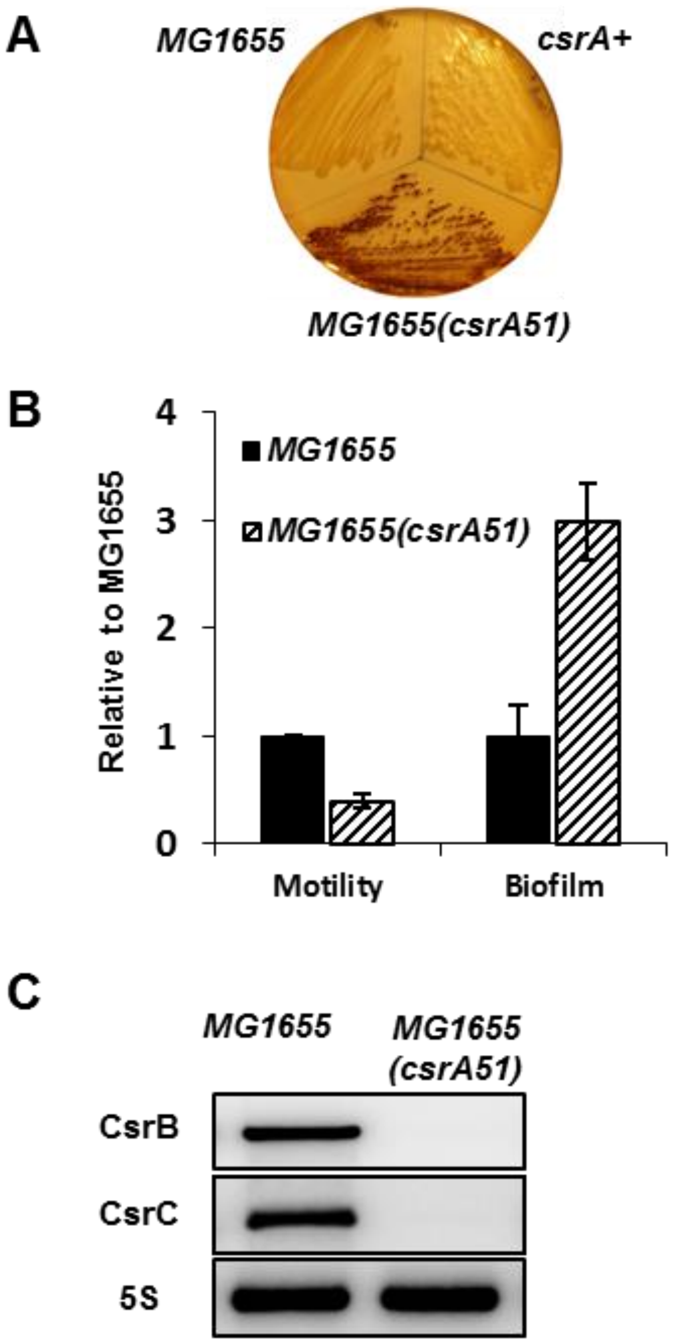

Figure S2. Effect of the *ΔcsrD* mutation on the stability of CsrB and CsrC sRNAs. CsrB and CsrC stability in MG1655 and MG1655(*ΔcsrD*) strains was measured in Northern blot and quantified by PhosphorImager. All experiments were repeated at least twice.

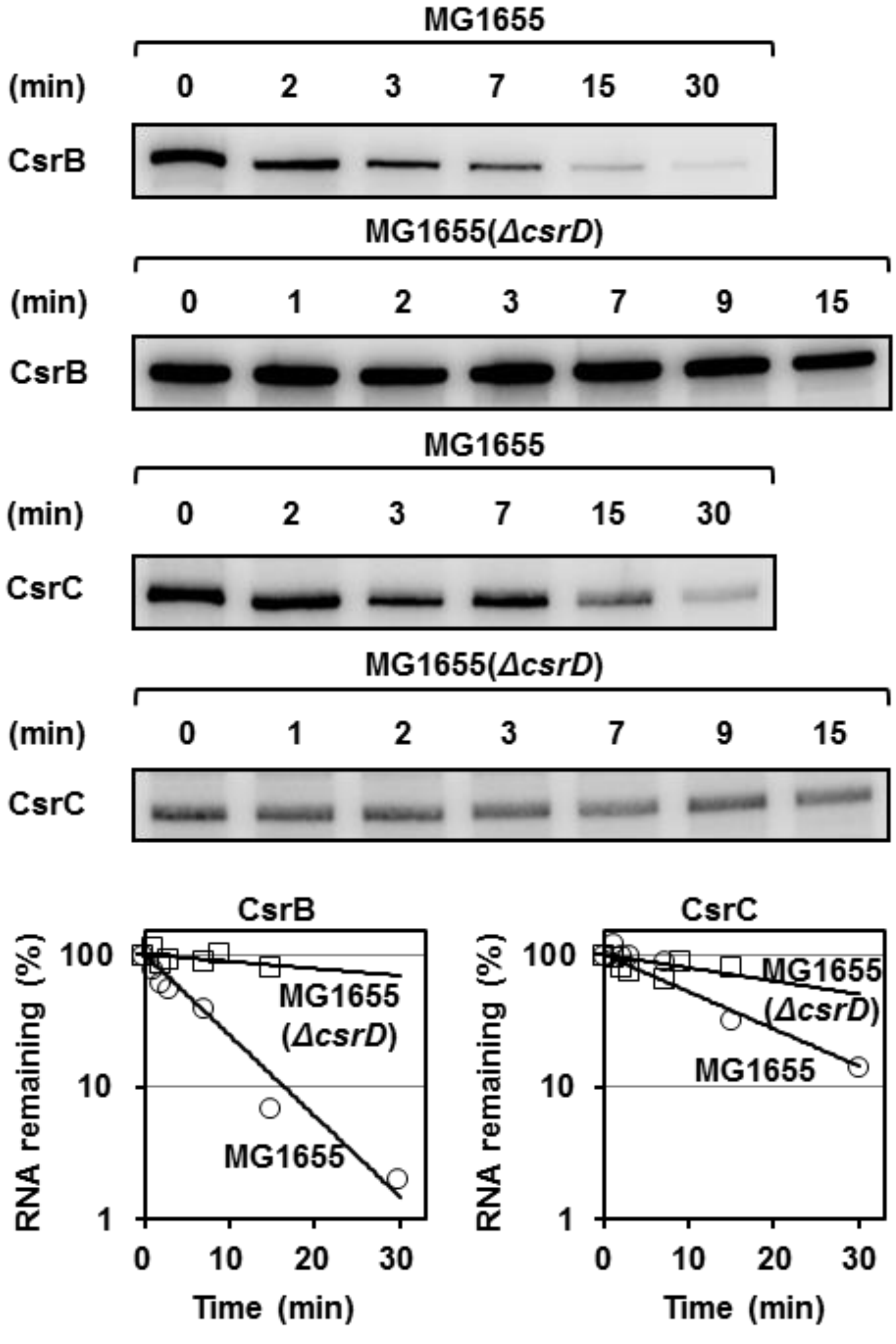

Table S4: Comparison of β-galactosidase activity of transcriptional fusions in the strains MG1655, MG1655(*csrA51*) and MG1655(*ΔcsrD*). Ratios of β-galactosidase activity between two strains are given.

	MG1655(*csrA51*) / MG1655		MG1655(ΔcsrD) / MG1655
glgB::lacZ	1.55 ± 0.13	csrC::lacZ	0.48 ± 0.00
ydeH::lacZ	1.34 ± 0.32	fdrA::lacZ	0.44 ± 0.03

Table S7. List of the 99 mRNAs for which direct regulation of stability by CsrA had an impact on quantity.

CsrA regulation: (-) negative and (+) positive in the MG1655 cells
Degradational coefficient ρD value: if ρD ≥ 0,6 (bold), mRNA amount is mainly controlled by degradation; if ρD ≤ 0,6 (italic) mRNA amount is equally controlled by degradation and transcription or mainly controlled by transcription

**Blattner**	**Gene Symbol**	**CsrA regulation**	**Fold change of half-lives MG1655(*csrA51)* / MG1655**	**Fold change of mRNA amounts MG1655(*csrA51)* / MG1655**	**Degradational coefficient ρ_D value**
b0072	*leuC*	-	1.50	1.28	**1.67**
b0073	*leuB*	-	1.53	1.34	**1.45**
b0118	*acnB*	-	2.15	1.12	**6.83**
b0212	*gloB*	+	0.69	0.65	**0.88**
b0391	*yaiE*	+	0.68	0.62	**0.82**
b0406	*tgt*	+	0.54	0.82	**3.01**
b0476	*aes*	+	0.72	0.71	**0.99**
b0688	*pgm*	-	1.41	3.19	*0.29*
b0721	*sdhC*	-	1.17	1.13	**1.28**
b0897	*ycaC*	+	0.29	0.61	**2.54**
b1042	*csgA*	+	0.46	0.77	**3.04**
b1089	*rpmF*	+	0.63	0.79	**1.97**
b1092	*fabD*	-	1.40	1.85	0.55
b1095	*fabF*	-	1.61	1.28	**1.94**
b1178	*pliG*	+	0.56	0.65	**1.33**
b1219	*ychN*	+	0.72	0.58	**0.60**
b1324	*tpx*	+	0.54	0.77	**2.31**
b1380	*ldhA*	+	0.60	0.82	**2.62**
b1415	*aldA*	+	0.65	0.93	**5.58**
b1440	*ydcS*	+	0.78	0.50	*0.35*
b1461	*pptA*	+	0.53	0.61	**1.27**
b1479	*maeA*	-	1.41	1.32	**1.25**
b1538	*dcp*	+	0.79	0.78	**0.92**
b1602	*pntB*	-	1.55	1.32	**1.56**
b1603	*pntA*	-	1.54	1.21	**2.25**
b1612	*fumA*	-	1.32	1.12	**2.48**
b1619	*hdhA*	+	0.64	0.77	**1.70**
b1667	*ydhR*	+	0.53	0.19	*0.38*
b1692	*ydiB*	+	0.57	0.64	**1.23**
b1694	*ydiF*	+	0.60	0.76	**1.84**
b1695	*ydiO*	+	0.65	0.73	**1.35**
b1718	*infC*	-	1.37	1.29	**1.23**

b1719	*thrS*	-	1.64	1.59	**1.06**
b1779	*gapA*	-	1.66	1.39	**1.54**
b1782	*mipA*	-	1.41	1.36	**1.12**
b1800	*dmlA*	+	0.69	0.67	**0.92**
b1817	*manX*	-	2.02	1.71	**1.32**
b1818	*manY*	-	2.29	1.25	**3.74**
b1819	*manZ*	-	2.10	1.37	**2.37**
b1823	*cspC*	+	0.64	0.28	*0.35*
b1824	*yobF*	+	0.45	0.37	**0.79**
b1852	*zwf*	+	0.79	0.93	**3.19**
b1901	*araF*	+	0.54	0.51	**0.90**
b2001	*yeeR*	+	0.60	0.06	*0.18*
b2094	*gatA*	-	1.62	1.19	**2.79**
b2095	*gatZ*	-	1.84	1.22	**3.06**
b2245	*yfaU*	+	0.57	0.82	**2.96**
b2246	*rhmT*	+	0.48	0.69	**2.02**
b2360	*yfdQ*	+	0.68	0.49	*0.55*
b2463	*maeB*	-	2.26	1.40	**2.43**
b2521	*sseA*	-	1.32	3.10	*0.24*
b2542	*hcaD*	+	0.73	0.84	**1.84**
b2702	*srlA*	+	0.31	0.53	**1.83**
b2703	*srlE*	+	0.28	0.42	**1.47**
b2704	*srlB*	+	0.32	0.39	**1.21**
b2799	*fucO*	-	2.96	4.07	**0.77**
b2937	*speB*	+	0.53	0.73	**2.02**
b2938	*speA*	-	1.49	1.19	**2.27**
b2989	*yghU*	+	0.59	0.65	**1.23**
b3008	*metC*	+	0.64	0.73	**1.41**
b3011	*yqhD*	+	0.50	0.72	**2.12**
b3067	*rpoD*	-	2.79	1.26	**4.37**
b3077	*ebgC*	+	0.57	0.66	**1.34**
b3078	*ygjI*	+	0.52	0.68	**1.69**
b3089	*sstT*	-	1.35	2.32	*0.36*
b3091	*uxaA*	+	0.66	0.80	**1.86**
b3092	*uxaC*	+	0.52	0.80	**2.93**
b3133	*agaV*	+	0.67	0.70	**1.10**
b3134	*agaW*	+	0.73	0.75	**1.12**
b3253	*yhdH*	+	0.69	0.65	**0.87**
b3310	*rplN*	-	1.29	1.13	**2.06**
b3313	*rplP*	-	2.10	1.17	**4.63**
b3316	*rpsS*	-	1.93	1.11	**6.10**
b3371	*frlB*	+	0.59	0.67	**1.30**
b3403	*pck*	-	2.61	1.25	**4.35**
b3432	*glgB*	-	1.92	3.58	*0.51*
b3433	*asd*	-	1.47	1.84	**0.63**
b3460	*livJ*	+	0.20	0.48	**2.20**

b3491	*yhiM*	+	0.57	0.76	**2.05**
b3774	*ilvC*	+	0.82	0.80	**0.88**
b3831	*udp*	+	0.51	0.63	**1.47**
b3870	*glnA*	-	1.71	1.33	**1.88**
b3919	*tpiA*	+	0.83	0.76	**0.71**
b3926	*glpK*	+	0.65	0.41	*0.49*
b3927	*glpF*	+	0.57	0.28	**0.44**
b3981	*secE*	-	1.34	1.35	**0.98**
b3990	*thiH*	+	0.55	0.87	**4.30**
b4034	*malE*	+	0.51	0.59	**1.26**
b4079	*fdhF*	+	0.75	0.65	**0.65**
b4088	*alsB*	+	0.66	0.62	**0.86**
b4120	*melB*	+	0.66	0.76	**1.53**
b4138	*dcuA*	-	1.99	4.33	*0.47*
b4211	*qorB*	+	0.67	0.83	**2.17**
b4216	*ytfJ*	+	0.54	0.66	**1.48**
b4234	*yjgA*	+	0.55	0.82	**2.99**
b4265	*idnT*	+	0.64	0.86	**2.96**
b4266	*idnO*	+	0.67	0.46	*0.51*
b4267	*idnD*	+	0.61	0.56	**0.86**
b4525	*ymjC*	+	0.59	0.87	**3.89**

Table S8. DNA oligonucleotides used in this study.

**Primer Name**	**Sequence** (5’ to 3’)
OKT-24	TGAGGGTGCGTCTCACCGATAAAGATGAGACGCGGAAAGACTTGAGCGATTGTGTAGGCT
OKT-25	GAAGGAAGTTTCTGTTCACCGTGAAGAGATCTACCAGCGTTAATCCATCCAGTCTATTAT
OCT-29	GGAGGTCTGACAGATAGTAG
OCT-30	TCACGGCATTCGCATTAACG
OCT-50	GCGTTAAAGGACACCTCCAGG
OCT-51	GTAATACGACTCACTATAGGTTCGTTTCGCAGCATTCCAG
OCT-52	GAGGACGCTAACAGGAACAATG
OCT-53	GTAATACGACTCACTATAGGTCTTACAATCCTTGCAGGC
MBO-59	ACTACCATCGGCGCTACGGC
MBO-139	TTACTTAGGATCCCCGACGTCGCGATTATACCTGAAC
MBO-140	CCTGTCCGGTGCCCTCTATGGGTGCTACTTTACGCC
MBO-197	TTACTTAGGATCCCCGCGCTATTCTAACGAGAGAAAAGC
MBO-198	CCTGTCCGGTGCCCTCGCGGTAATTTGATTTCTCACAAAG
MBO-199	GGGGATCCTAAGTAAGTAAGGAG
MBO-200	AGGGCACCGGACAGGTCG
MBO-203	TTACTTAGGATCCCCCCTTAAAGAATAGCCAATGCTCTATTTAAC
MBO-204	CCTGTCCGGTGCCCTTCCTGTCAGCCTTTACCGTGG
MBO-207	TTACTTAGGATCCCCATTCCACTGCTCCTTTATAGGTAC
MBO-208	CCTGTCCGGTGCCCTGCAACGCGACGGCGAGAC
